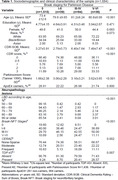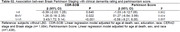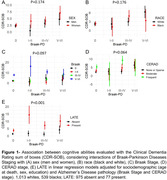# Lewy body disease stages through aging and correlations with clinical and neuropathological profile on a population‐based brain bank

**DOI:** 10.1002/alz.092753

**Published:** 2025-01-03

**Authors:** Vitor Ribeiro Paes, Alberto Fernando Oliveira Justo, Roberta Diehl Rodriguez, Michel Satya Naslavsky, Renata Elaine Paraizo Leite, Carlos Augusto Pasqualucci, Mayana Zatz, Wilson Jacob‐Filho, Claudia Kimie Suemoto, Lea T. Grinberg

**Affiliations:** ^1^ Physiopathology in Aging Laboratory (LIM‐22), University of Sao Paulo Medical School, São Paulo, São Paulo Brazil; ^2^ University of São Paulo Medical School, São Paulo, São Paulo Brazil; ^3^ Human Genome and Stem Cell Center, São Paulo, São Paulo Brazil; ^4^ Biobank for aging studies of the University of São Paulo, São Paulo Brazil; ^5^ Physiopathology in Aging Laboratory (LIM‐22), University of São Paulo Medical School, São Paulo Brazil; ^6^ Division of Geriatrics, University of São Paulo Medical School, São Paulo Brazil; ^7^ Division of Geriatrics, Department of Internal Medicine, University of São Paulo Medical School, São Paulo, São Paulo Brazil; ^8^ Memory & Aging Center, Department of Neurology, University of California in San Francisco, San Francisco, CA USA

## Abstract

**Background:**

Lewy body disease (LBD) is a neurodegenerative condition marked by the accumulation of neuronal alpha‐synuclein, leading to both Parkinson’s disease and Lewy body dementia. It is regarded as the second most common neurodegenerative disease associated with aging. However, there is limited knowledge of LBD prevalence in the general population, particularly among non‐whites. Our study aimed to investigate the prevalence of LBD in a large, diverse population‐based clinicopathological cohort. Additionally, we sought to explore the associations of LBD with comorbid neuropathology, clinical outcomes, and APOE genotyping.

**Method:**

We investigated cases from the Biobank for Aging Studies from the University of São Paulo, collected between 2004 and 2023. Clinical and sociodemographic information were gathered from the next of kin using structured and validated protocols, including parkinsonism score (PS) assessment through Tanner’s questionnaire. Neuropathological evaluations adhered to USA/NACC guidelines, using immunohistochemistry to detect beta‐amyloid, tau, alpha‐synuclein, and TDP43.

**Results:**

1,669 participants with complete sociodemographic, clinical, and neuropathological data were enrolled. LBD was detected in 0,84% of 50‐59 age group and the frequency increased with age reaching 15% in 90+ group. There was a trend of lower LBD presence in Black/Brown, but higher in Asians. LBD was associated with age, and higher CDR‐SOB and PS scores (Table 1). LBD prevalence increased with worse Braak‐NFT, CERAD, and LATE. The presence of LBD was associated with CDR‐SOB score in Braak PD stages ≥ III. However, Braak PD scores were not related to PS scores in adjusted analyses (Table 2). LBD interacted with LATE, CERAD, and Braak‐NFT on cognitive scores (Figure 1); no difference was observed in interaction with sex and race. Upon conducting a stratified analysis, LATE had a significant impact on cognitive abilities and LBD presence in limbic‐neocortical reas (ß = 2.30, 95%IC = 0.61; 8.59, p = 0.024).

**Conclusion:**

In an admixed population, LBD is present since middle age. The trend of less frequent LBD in Black/Brown warrants further investigations. LBD was associated with higher CDR‐SOB scores in neocortical phases (V‐VI) and more pronounced in participants with LATE.